# Taking knowledge users’ knowledge needs into account in health: an evidence synthesis framework

**DOI:** 10.1093/heapol/czv079

**Published:** 2015-08-31

**Authors:** Deepthi Wickremasinghe, Shyama Kuruvilla, Nicholas Mays, Bilal Iqbal Avan

**Affiliations:** ^1^London School of Hygiene & Tropical Medicine, London, UK and; ^2^World Health Organization, Geneva, Switzerland

**Keywords:** Communication research into policy, evidence into policy, knowledge

## Abstract

The increased demand for evidence-based practice in health policy in recent years has provoked a parallel increase in diverse evidence-based outputs designed to translate knowledge from researchers to policy makers and practitioners. Such knowledge translation ideally creates user-friendly outputs, tailored to meet information needs in a particular context for a particular audience. Yet matching users’ knowledge needs to the most suitable output can be challenging. We have developed an evidence synthesis framework to help knowledge users, brokers, commissioners and producers decide which type of output offers the best ‘fit’ between ‘need’ and ‘response’. We conducted a four-strand literature search for characteristics and methods of evidence synthesis outputs using databases of peer reviewed literature, specific journals, grey literature and references in relevant documents. Eight experts in synthesis designed to get research into policy and practice were also consulted to hone issues for consideration and ascertain key studies. In all, 24 documents were included in the literature review. From these we identified essential characteristics to consider when planning an output—Readability, Relevance, Rigour and Resources—which we then used to develop a process for matching users’ knowledge needs with an appropriate evidence synthesis output. We also identified 10 distinct evidence synthesis outputs, classifying them in the evidence synthesis framework under four domains: key features, utility, technical characteristics and resources, and in relation to six primary audience groups—professionals, practitioners, researchers, academics, advocates and policy makers. Users’ knowledge needs vary and meeting them successfully requires collaborative planning. The Framework should facilitate a more systematic assessment of the balance of essential characteristics required to select the best output for the purpose.

**Key Messages**

- The increased demand for evidence-based health policy in recent years has provoked a parallel increase in diverse evidence-based outputs designed to translate knowledge from researchers to policy makers and practitioners, yet matching users’ specific knowledge needs to the most suitable output, while essential, can be challenging.
- We have developed an evidence synthesis framework classifying 10 distinct evidence synthesis outputs under four domains: key features, utility, technical characteristics and resources, in relation to six primary groups of users—professionals, practitioners, researchers, academics, advocates and policy makers.
- We propose a process for matching users’ knowledge needs with an appropriate evidence synthesis output, using essential characteristics to consider when planning an output—Readability, Relevance, Rigour and Resources.
- When used in combination, the framework and process should facilitate a more systematic assessment of the balance of essential characteristics required to select the best output for the purpose and help knowledge users, brokers, commissioners and producers decide the best ‘fit’ between ‘need’ and ‘response’.

## Introduction

Increasing demands for the use of knowledge to assist evidence-based practice have led to a bourgeoning of different responses from funders and academics to evidence synthesis designed to support knowledge translation (Hansen and Rieper 2009). Each synthesis method and the type of output produced has its own merits and fulfils a particular knowledge need, for a particular primary audience, in a particular context. There are a number of factors that need to be considered when planning an evidence synthesis output including timeliness, length and format and the type of information to be included—whether solely research-based information, or the views of experts in the field, or a *hybrid* of both (Ogilvie *et al*. 2009; Abrami *et al.* 2010).

A diverse range of evidence synthesis outputs has been developed to meet users’ knowledge needs, including evidence articles, evidence briefs, knowledge summaries and systematic reviews. Yet identifying the most suitable evidence synthesis method and type of output for a particular need may be far from straightforward. One reason for this is that the labels given to different forms of output are not standardized, leaving scope for misunderstanding when commissioning and designing such reports (Arksey and O’Malley 2005; Grant and Booth 2009).

Each potential audience has different knowledge needs and the evidence may need to be presented in different ways to enhance its utility. Based on the opinions of an expert panel, we focus on six primary audiences: researchers, academics (who may also be researchers), advocates (largely those working for non-governmental organizations, NGOs), policy makers, administrative and managerial professionals, and practitioners. (The latter two groups are concerned with policy implementation, through delivering services and may also include NGO workers). Each of these groups requires knowledge for different purposes (Table 1). Evidence syntheses may have mutliple users and be used at mutliple levels of the health system. The audience groups that we have not addressed are considered in the discussion section, as one of the study’s limitations.

**Table 1.** Users’ knowledge needs

Academics and researchers	Advocates	Policy makers	Professionals and practitioners
To critically appraise new and exisiting research and identify gaps in research, to both verify and generate knowledge	To have an overview of research with illustrative evidence-based case studies to inform advocacy for changes in policy and practice	To gain an understanding of validated concepts, experiences and technical knowledge on which to develop new or change existing policy	To have access to validated concepts, experiences and technical knowledge to assist with implementing policy and best practice

This study aims to contribute to an understanding of different users’ knowledge needs and how they can be met through matching them with relevant evidence synthesis outputs. The objectives are to identify: different evidence synthesis outputs and their distinguishing features; as well as issues to consider when planning the development of an evidence synthesis to match users’ knowledge needs.

We have created an evidence synthesis framework describing the features, benefits and limitations of outputs, based on a literature search, and consultations and interviews with experts in the field of synthesizing research for policy and practice. This framework should benefit both commissioners and producers of synthesis outputs—including knowledge brokers, who are responsible for deciding which type of output will best meet the needs of the evidence users they support.

The scope of this study is the wide range of diverse evidence synthesis outputs, which encompasses, but is not exclusive to systematic reviews. Much of the existing literature focuses on methodologies to analyse quantitative and/or qualitative studies that are variants of systematic reviews, e.g. Gough and Elbourne 2002; Mays *et al.* 2005; Tricco *et al*. 2011; Hansen and Rieper 2009. These are well-defined, distinct approaches (e.g. meta-analysis, or realist, diagnostic test or complex reviews etc.). However in this study, the nature of systematic reviews is acknowledged as a generic type of evidence synthesis output.

## Methods

A four-strand literature search, described below, was conducted to ascertain what research exists that contributes to answering the study objectives. Using the methodology for a systematic review was not feasible because of the nature of the documents on which the literature search was based. Such documents, for policy makers and a general audience are not generally found in databases of academic peer-reviewed articles. Nevertheless, the methodology we used followed parameters which were intended to make it systematic.

The first strand of the literature search was a search of five bibliographic databases of peer-reviewed journal articles: Embase, Global Health, Medline, Social Policy & Practice and Web of Science. Based on the number of relevant articles from particular journals identified in the database search, the second strand was a hand search of three peer-reviewed journals that were considered particularly relevant: Systematic Reviews Journal; Journal of Health Services Research & Policy; and BMC Medical Research Methodology. The third strand was a search for relevant grey literature using Google. This was not exhaustive, but was as comprehensive as possible, representing five leading organizations involved in producing evidence syntheses: the UK Department for International Development (DFID), the Overseas Development Institute, INASP (an international development charity working with a global network of partners to improve access, production and use of research information and knowledge), the UK Economic and Social Research Council (ESRC) and the International Initiative for Impact Evaluation (3iE). DFID’s Research for Development (R4D) database was also searched.

The inclusion criteria for the literature search were that articles were written in English and were either review or discussion articles. The initial search terms used for the first two strands of the literature review were:

*evidence synthesis* (singular and plural) *AND methodology.*

A second search of the bibliographic databases was then undertaken using the search terms (*expert opinion OR consensus statement) AND policy making.*

**Figure 1.**
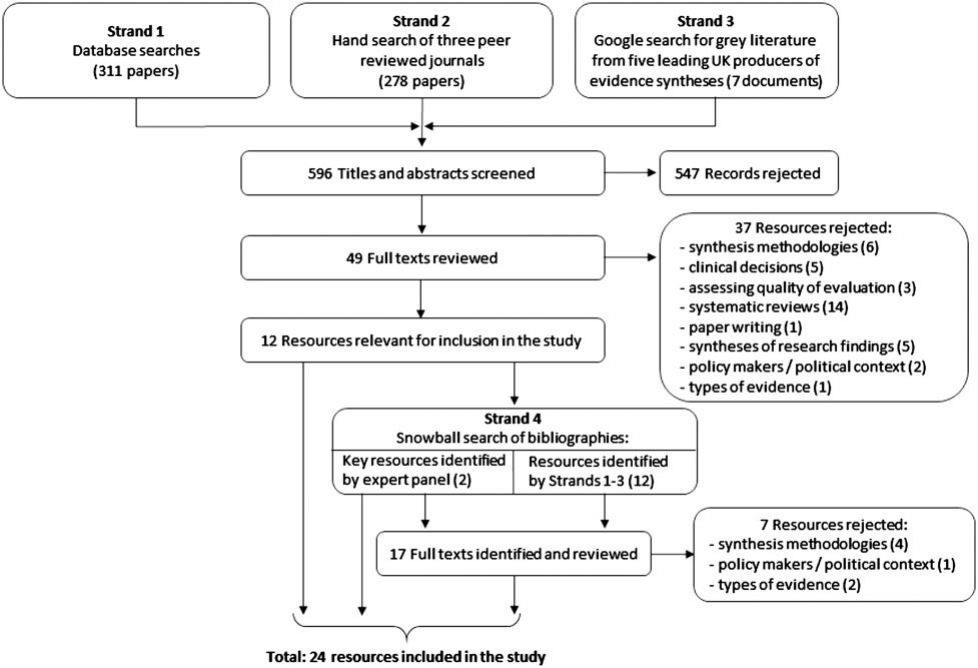
Flow diagram of literature search

Once the search results were compiled using Endnote, the titles and abstracts (or executive summaries) of all records were appraised and 49 were considered to be relevant. Given the small number of documents, one researcher read all 49 in full and made a decision as to whether or not they met the study objectives of identifying different types of evidence synthesis, or highlighted issues to consider when planning the development of an evidence synthesis to match users’ knowledge needs (Supplementary data 1 are available at *HEAPOL* online). Twelve documents were considered to meet these objectives. The fourth strand of the literature search was to use a snowball technique to identify further documents from the references cited in these 12 documents, as well as two key documents identified by the expert panel we consulted, bringing the total number of relevant documents to 24. Figure 1 shows a flow diagram of the literature search. One researcher conducted the literature search and the decisions made were reviewed with a second researcher at regular intervals.

Experts in synthesis designed to get research into policy and practice were also consulted to hone issues for consideration and ascertain key studies. We consulted with a panel of eight experts, from diverse backgrounds, with experience of producing evidence syntheses. They were selected purposively because they represented the various types of expertize needed to produce such outputs and included a leading research scientist involved in knowledge translation, health system researchers, advocacy and communications specialists and representatives from large organisations that regularly produce evidence synthesis outputs, and advisers to policy makers (Supplementary data 2 are available at *HEAPOL* online). Prior to the literature search, a discussion guide was devised to focus phone and face-to-face meetings with four of these experts. It included identifying the need for evidence syntheses, the value of a question-based evidence synthesis, the value of synthesized evidence versus expert opinion, sound examples of typologies of evidence syntheses and different types of evidence synthesis outputs and their relative validity.

These meetings developed into free-flowing discussion, providing insights and suggestions that helped to determine some of the essential characteristics of different types of evidence synthesis outputs. These discussions informed a manual synthesis of the literature search findings, from which a framework and report were developed with the participation of all eight experts, who gave useful feedback, particularly in fine-tuning the framework and recommendations.

## Results

**Figure 2.**
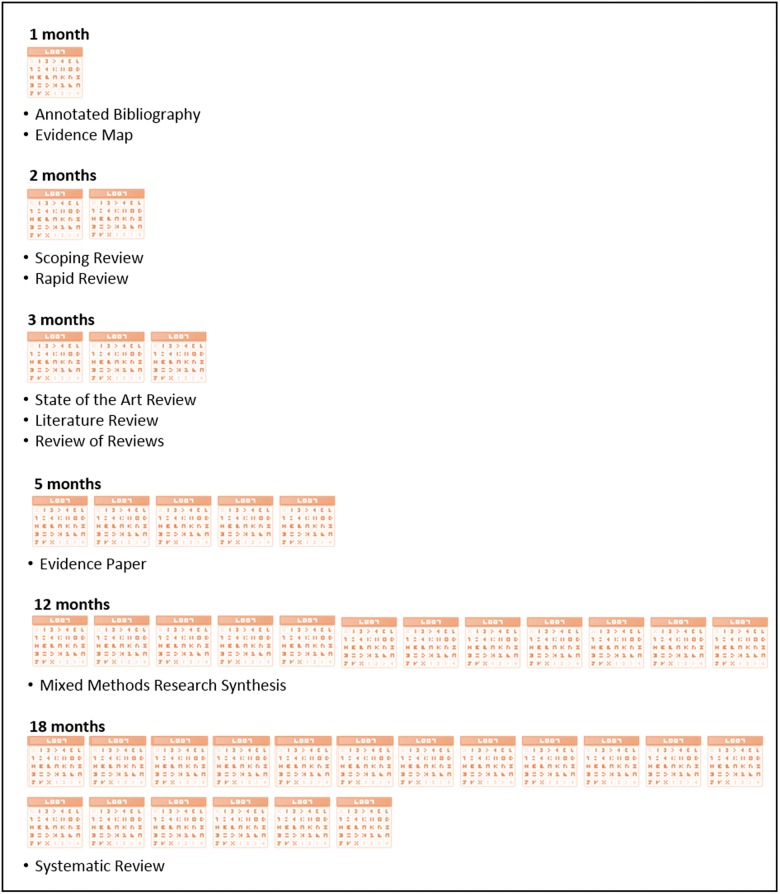
Resources: Indicative production times for evidence synthesis outputs

We identified 10 different forms of evidence synthesis outputs and have classified them in an evidence synthesis framework. The Framework arranges the characteristics of these outputs under four domains: there is a brief description of each output’s key features; its utility for the primary audience we suggest it is best suited to; technical characteristics, including limitations; (Tables 2–4) and the production resources that should be considered, in order to meet knowledge users’ needs, such as a timeframe (Figure 2).

**Table 2.** Evidence synthesis framework—key features of forms of evidence synthesis outputs

	Evidence synthesis outputs based on a broad thematic overview				Evidence synthesis outputs based on a specific question					
Commonly used name	Annotated bibliography	Evidence map	Scoping review	State of the art review	Rapid review	Literature review	Review of reviews	Evidence paper	Mixed methods research synthesis	Systematic review
Also known as		Mapping review	Critical review	Knowledge summary	Evidence summary	Overview	Umbrella review	Evidence briefing	Multi-arm systematic review	
		Systematic map	Scoping study		Rapid evidence assessment		Overview of reviews	Briefing note	Mixed studies review	
					Interim evidence assessment			Evidence to policy brief		
					Brief review			Evidence brief		
					Strategy brief			Research summary		
Description	A list of key literature and/or sources, primarily of research evidence with expanded summaries on the main content	A map of the existing research evidence base to provide an overview of key themes and/ or results and identify research gaps	An overview of research undertaken on a (constrained) topic, when time and other constraints are limited	A brief review primarily of recent research evidence	A quick review of key, easily accessible evidence, from research and other sources, on a particular (constrained) topic	An overview and synthesis primarily of research evidence with key conclusions	Includes existing reviews, preferably systematic rather than primary studies, and draws a conclusion statement	An extensive overview of available and accessible evidence—both peer reviewed and significant grey literature—primarily from research	A full map and synthesis of different types of research evidence—both quantitative and qualitative—to answer a research question and subquestions	An exhaustive and robust review and synthesis of research evidence
	Often produced for a specific, time bound purpose	Often produced for a specific, time bound purpose	Often produced for a specific, time bound purpose	May include a consensus statement drawing on practice-based evidence	Often produced for a specific, time bound purpose	Is likely to include a critical appraisal of research		Includes a balanced, objective assessment and critical appraisal of the evidence	May include statistical meta-analysis of quantitative medical research and a synthesis of qualitative data	Includes a map of evidence, critical appraisal and qualitative or quantitative evidence synthesis
						May give an indication of areas of consensus and debate		Includes a commentary on evidence	Mixed methods research syntheses include realist reviews and meta-narrative reviews	Includes the criteria (e.g. quality, date range, method) applied to select evidence for synthesis
						Includes peer-reviewed literature and is likely to include grey literature		May consider local context and cost effectiveness		Incorporates peer-reviewed and significant grey literature
										Draws a clear scientific conclusion

**Table 3.** Evidence synthesis framework—utility of different forms of evidence synthesis outputs for their primary audience

	Evidence synthesis outputs based on a broad thematic overview				Evidence synthesis outputs based on a specific question					
Commonly used name	Annotated bibliography	Evidence map	Scoping review	State of the art review	Rapid review	Literature review	Review of reviews	Evidence paper	Mixed methods research synthesis	Systematic review
Suggested primary audience	Researchers/academics	Researchers/academics	Researchers/academics	Advocates/ Policy makers	Policy makers	Researchers/academics	Researchers/academics	Professionals/practitioners	Professionals/practitioners	Professionals
When is it useful?	To identify documents that may have particular relevance to a topic	To give an overview of key issues and where or what evidence exists	To determine the range of studies that are available on a specific topic	To provide timely evidence to support advocacy for policy and practice	To provide a rapid overview of key issues and publications for a specific, immediate purpose (e.g. workshop input, speech, timely policy decisions, initial scoping)	To provide information on a specific topic in a short period of time	When there is a considerable body of research and a number of research reviews in a particular area	To set out a comprehensive evidence base sufficient to underpin policy decisions or programme designs	When a synthesis of both statistical and qualitative data are required, drawn from a wide range of sources	When time and resources are available, this provides the most comprehensive and authoritative summary of a body of evidence at a particular point in time, to underpin policy decisions or programme designs
	May complement other review outputs, particularly rapid reviews or evidence maps	May inform more in-depth reviews	To determine the value of undertaking a systematic review		To help identify key issues and/or questions for more in-depth reviews	To synthesize the existing evidence base as a guide for policy and programme decisions within a set timeframe		When time and/or fiscal resources are not available for a full systematic review	Provides a comprehensive and authoritative summary of a body of evidence at a particular point in time, to underpin policy decisions or programme designs	
			To summarize and disseminate research findings			To determine existing evidence and identify future evidence needs		May form the basis for a full systematic review		
			To identify research gaps in the existing literature			May direct or refine questions for more in-depth reviews				
Examples	http://www.cihr-irsc.gc.ca/e/40740.html	http://www.hindawi.com/journals/drt/2012/820735/	http://www.ncbi.nlm.nih.gov/pmc/articles/PMC3128401/	http://www.who.int/pmnch/knowledge/publications/summaries/ks27/en/index.html	http://www.who.int/pmnch/media/events/2013/au_policy_brief_aids_tb_malaria.pdf?ua = 1	http://www.health.vic.gov.au/agedcare/maintaining/downloads/healthy_litreview.pdf	http://www.who.int/pmnch/topics/part_publications/essential_interventions_18_01_2012.pdf	http://www.who.int/pmnch/topics/economics/costoolsreviewpack.pdf?ua = 1	http://www.physiotherapyuk.org.uk/visiting/programme/presentations/2199	http://www.globalizationandhealth.com/content/9/1/15
								http://www.who.int/pmnch/knowledge/publications/2011_accountability-mechanisms/en/

**Table 4.** Evidence synthesis framework—technical characteristics of different forms of evidence synthesis outputs

	Evidence synthesis outputs based on a broad thematic overview				Evidence synthesis outputs based on a specific question					
Commonly used name	Annotated bibliography	Evidence map	Scoping review	State of the art review	Rapid review	Literature review	Review of reviews	Evidence paper	Mixed methods research synthesis	Systematic review
Quality appraisal of evidence	Limited	Limited	Limited	Limited	Limited	Limited	Essential	Essential	Essential	Essential
Evidence usually presented as	Reference list	Graphics and tables	Narrative and tables	Narrative, graphics and tables	Narrative and tables	Narrative	Narrative, graphics and tables	Narrative and tables	Narrative, graphics and tables	Narrative and tables
Systematic documentation of evidence	Limited	Comprehensive	Limited	Limited	Comprehensive	Comprehensive	Comprehensive	Comprehensive	Comprehensive	Comprehensive
Replicable	Low	Medium	Low	Low	Medium	Medium	Medium	Low	Medium	High
Periodic update	Possible	Possible	Possible	Possible	Possible	Possible	Essential	Possible	Possible	Essential
Limitations	Does not synthesize or analyse findings across sources	Overview, not in-depth analysis	May have: A narrow focus question	-Evidence base not comprehensive, limited to most recent scientific information	Evidence base not comprehensive	Prone to selection and publication bias - tends to review readily available evidence	Does not include research outside existing reviews	Limited accessibility to literature	Time consuming and resource intensive	Resource intensive (time, human, financial)
	Generally does not appraise evidence	Does not synthesize or analyse findings across sources	Few search sources	May be prone to bias	Relies on easily accessible/ available evidence	Often limited detail on search strategies, or how conclusions reached	Because reviews are of variable quality, each needs to be assessed for how systematic and comprehensive it is	Time/human resource constraints likely to limit scope		May have a narrow clinical question or set of questions
	Prone to selection and publication bias	A range of evidence may be covered, but generally relies on few search sources	Use only key terms for search (not all variants)		Prone to selection and publication bias	Resources determine scope, which may limit comprehensive-ness or lead to inconclusive findings		Limited literature search		Has a history of use in health and education; yet to be fully tested in other development areas, e.g. governance and climate change
		Prone to selection and publication bias	Be limited to electronic and easily available documents		Risk of generating inconclusive findings that provide a weak answer to the original question					
	;		A simple description with limited analysis							

### Different forms of evidence synthesis outputs and their distinguishing features

These outputs synthesize different types of evidence; some include evidence outside that produced by scientific research. Hansen and Rieper (2009) observe the rise of evidence-based policy making and delivery in Europe since the 1990s and differentiate between the forms of evidence used, based on Eraut’s (2004) work on the credibility of evidence used for decision making. Eraut distinguishes between research-based evidence in peer-reviewed published research; other scientific evidence (generated using scientific procedures with a track record of producing valid results); and practice-based evidence (derived from recognized professional practices that have been undertaken using criteria expected by experts within the profession). Any, or all of these could make a valid and useful contribution, but may not in themselves be sufficient to meet policy makers’ needs.(Mays *et al*. 2005) The Partnership for Maternal, Newborn and Child Health (PMNCH) strategy briefs (2014b) are an example of practice-based evidence syntheses combined with tools to develop and implement strategies to inform advocacy, policy and practices.

We found a number of studies that describe some of the different evidence synthesis outputs in similar terms, and these have contributed to the development of the evidence synthesis framework, yet none covers all four domains. For example, to help commissioners identify which evidence synthesis output would best suit a particular need, the UK Civil Service (2010) and DFID (2013) suggest when an output might be useful and its limitations, but neither includes many technical characteristics. Other frameworks are based on synthesis methods, but do not take users’ perspectives or the resources required into account. Grant and Booth (2009) present a comparison framework based on the four main processes used to review evidence—Search, AppraisaL, Synthesis and Analysis (SALSA)—to distinguish between different syntheses and define their characteristics. Classification differences mean that some of the outputs they identify share a definition in the Framework we have developed. Kastner *et al.* (2012) also map the characteristics of existing evidence synthesis methods, and Tricco *et al.* (2011) use the qualitative or quantitative nature of sources of evidence to tabulate the characteristics of different synthesis methods, which they refer to as ‘…*types of systematic reviews’.*

Other studies focus on evidence synthesis outputs guided by a clear question and primarily synthesizing research evidence, and present methodological frameworks based on the type of research question to which an answer is sought (Petticrew and Roberts 2003; Mays *et al*. 2005). The need for an evidence synthesis to have a research or learning question came up repeatedly in the literature search and was discussed with the expert panel. A carefully structured research or learning question can help to clarify and target the literature search and places the synthesis within a context, including a theoretical context (Gough and Elbourne 2002) and some consider that it guides the whole production process (Gough and Elbourne 2002; Petticrew and Roberts 2003; Mays *et al.* 2005; DFID Evidence Brokers 2013).

For researchers and practitioners, who are generally concerned with impact and effectiveness issues, well-established outputs that are primarily based on research studies—such as systematic reviews—are designed to answer specific impact questions, e.g. *What evidence is there that misoprostol can prevent postpartum haemorrhage?* Although the knowledge to action (KTA) evidence summaries prepared as part of a collaborative project between the Champlain Local Health Integration Network and the University of Ottawa, funded by Canadian Institutes of Health Research, were not initially developed with a predetermined primary research question, user feedback suggested it would provide *‘clarity and direction’* (Khangura *et al*. 2012). An iterative process was built into future summaries, so that the research team worked with users to agree a research question. Similarly, Chambers and Wilson (2012) propose a checklist by which researchers and users’ representatives, or commissioners can clarify the research question.

UK Civil Service guidelines (2010) group evidence that can be synthesized around *non-impact questions* e.g. needs, process, implementation, correlation, attitude and economic questions, such as; *How much does it cost to deliver misoprostol to pregnant women in community settings?* Yet a research question may not be a key requirement for all knowledge users; for some, a more general focus might be appropriate. Advocates, policy makers and implementers may have a variety of issues to consider and require a range of evidence beyond scientific research, to guide them (Sheldon 2005; Lavis *et al*. 2009; Abrami et al. 2010). Davies (2006) notes that policy makers often want answers to broad questions, which may not always be sufficiently focussed to guide a tight search for evidence beyond that available from research; *‘such as administrative data and evidence used by lobbyists, pressure groups and think tanks (which may or may not be research based)’.* While there are a limited number of databases available to help guide such searches, e.g. Open Grey, these are not exhaustive and often have a basic search function. A clear statement of the issue might be a more suitable starting point (Gough and Elbourne 2002; Petticrew *et al*. 2004; Mays *et al.* 2005; UK Civil Service 2010; Chambers and Wilson 2012; Khangura *et al.* 2012) as in the PMNCH knowledge summaries (2014a). Our evidence synthesis framework distinguishes between those evidence synthesis outputs which address a specific research question and those which provide a broad thematic overview of the evidence relating to issues in a policy area, such as significance, as in the PMNCH knowledge summary *Maternal mental health: Why it matters and what countries with limited resources can do* (Hashmi 2014).

Variations in the names and characteristics of some types of evidence synthesis outputs meant that categorizing them in the Framework was not always straightforward. For example, the Alliance for Health Policy and Systems Research (2011) splits synthesis outputs into short syntheses and systematic reviews, noting that the names of short synthesis documents—policy brief, research summary and briefing note, ‘*…are typically used indiscriminately, and could refer to similar or highly dissimilar ideas’*. It reclassifies short synthesis outputs, by the type and extent of the information they summarize.

While standardizing the names and methods would help clarify and distinguish between outputs with partially or fully overlapping characteristics, some researchers consider this unnecessary or even restrictive, suggesting that a preferable solution would be to include a transparent statement of methods in each output (Gough and Elbourne 2002; Watt *et al*. 2008; Ganann *et al.* 2010). The Effective Health Care bulletins, commissioned by the English Department of Health, are one example where methodological information is included (Centre for Reviews and Dissemination 2004). Another is the evolution of evidence summaries produced under the KTA research programme, where iterative feedback from users of early summaries led to the development of a template that includes a methods section (Khangura *et al*. 2012).

### Factors to consider when planning an evidence synthesis output

Planning an evidence synthesis ideally involves collaboration between those commissioning and those producing an output. The challenge is to ensure that it meets the users’ specific information needs, is user-friendly, timely and credible (Sheldon 2005). Consideration of some essential characteristics should help. When offering guidance to researchers writing for a diverse audience, Largay (2001) identifies *Three Rs*—Readability, Relevance and Rigour as essential characteristics. **Rigour** relates to the systematic and transparent application and recording of the method used. **Relevance** refers to planning the scope of the evidence synthesis to fit the knowledge requirements of potential users, ensuring timely production and identifying the primary audience—why the research topic is important to them and what the context is. **Readability** includes using plain, non-technical language, clarity of thought and a brief summary or visual display of the conclusions reached.

**Figure 3.**
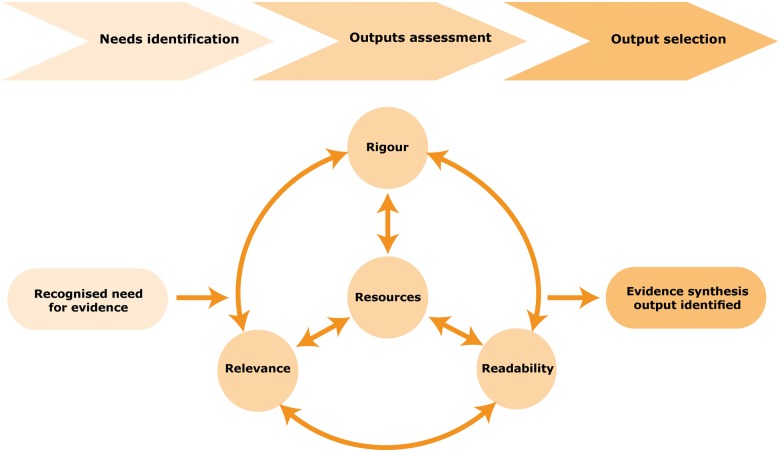
Process for matching information needs with an evidence synthesis output

Considering the three Rs should help secure a credible, timely and appropriate output that meets users’ needs. Grant and Booth (2009) and Thomson (2013) highlight a tension between rigour and relevance, given that the opportunities for using an output, for example within a defined policy window, may not allow sufficient time to undertake a systematic review. To help address this, Thomson (2013) considers the Three Rs as *‘interrelated principles’* that can be applied to planning evidence syntheses, particularly complex reviews to support policy making, and suggests they are considered in relation to a fourth R—**Resources** available for production (including time, funding and personnel). This helps determine a feasible and relevant scope for the synthesis output within the time available. Building on Thomson’s concept, Figure 3 shows how the *Four Rs* fit into a process for matching information needs with appropriate evidence synthesis outputs: once the need for synthesized evidence has been established, an acceptable balance between the Four Rs is agreed and used to make an objective assessment of the types of evidence synthesis outputs, to help identify the most appropriate output.

Relevance often relates to the particular context in which evidence synthesis outputs are to be used (Petticrew *et al*. 2004; Sheldon 2005; Ogilvie *et al*. 2009; Chambers and Wilson 2012; Saul *et al.* 2013). Researchers and producers of evidence syntheses need to develop some understanding of the knowledge needs of the primary audience and the environment in which they are working so as to analyse and present the information in a way that is relevant and helpful to users (Sheldon 2005). Such factors may relate to context, cost effectiveness and expert—or even public—opinion (Ogilvie *et al*. 2009), e.g. PMNCH strategy briefs (2014b) are often produced in more than one language and use regional case studies, to support international or regional meetings.

A study eliciting the views of UK policy makers on how research evidence influences public health policy found that the attributes of evidence synthesis they considered to be important were broadly in line with three of the four Rs: clarity, timeliness and relevance to current policy debates, with the addition of attending to evidence of cost-effectiveness (Petticrew *et al*. 2004). In some instances, the inclusion of different types of evidence drawing on a wide range of information sources may be best suited to the production of a *hybrid* output that offers a peer-reviewed synthesis of recent scientific evidence with practical information for policy makers and practitioners (Abrami *et al*. 2010), such as the PMNCH knowledge summaries (2014a).

The relationship between the relevance of a synthesis output and the resources available to ensure its timeliness is an important planning consideration (Saul *et al*. 2013; Thomson 2013). Figure 2 gives indicative average production times for each of the evidence synthesis outputs in the evidence synthesis framework. Consideration of this and other resource issues by both commissioners and producers will likely affect various aspects of an output, including its rigour, depth, quality appraisal and scope. For example, resources generally influence the number of reviewers who can be employed to work on an output in the time available. Abrami *et al.* (2010) make this distinction clear by using *brief review* to describe a synthesis limited in both timeframe and scope, and *comprehensive review*, for one which is time bound, but not limited in scope because a number of researchers can work on it.

## Discussion

The Framework identifies 10 different forms of evidence synthesis outputs drawn from the literature search and consultation with experts. It shows the range of outputs that have been developed in recent years to accommodate different evidence needs, beyond clinical decision making. Given the confusion produced by the many different terms used in the literature to describe these various forms of evidence synthesis outputs, the Framework, used in conjunction with the process for matching users’ information needs with an appropriate evidence synthesis output, is intended to offer greater clarity to users, commissioners and producers of outputs.

Using the process outlined in Figure 3, in conjunction with the evidence synthesis framework, offers a more systematic approach than was previously available to planning an appropriate evidence synthesis output by ensuring that all the essential features and characteristics, including resources, are considered. If planning is an iterative and participatory collaboration between users and/or commissioners and the production team, it will be a significant contributing factor towards producing an output tailored to meet users’ knowledge needs (Watt *et al.* 2008; Khangura *et al*. 2012; Saul *et al*. 2013) and increase the prospect of research being used in policy development (Corluka *et al*. 2014). Once the need for an evidence synthesis has been identified, those commissioning it should consider what sorts of evidence would be relevant and the level of rigour with which the evidence needs to be analysed for the particular context in which the synthesis will be used. In addition, the level of knowledge and understanding of the end-users needs to be appraised, to guide the level of technical language and detail that is required. Alongside these considerations, the resources available for production should also be taken into account. Taking the decisions made on relevance, rigour, readability and resources a match can then be made using the outputs listed in the Framework and the indicative average production times, in order to identify the most suitable output.

The strength of our approach was that we consulted with specialists in this field to guide the focus of the evidence synthesis framework and the process for matching users’ information needs with appropriate evidence synthesis outputs, but we acknowledge that in this field other perspectives on the issues considered may exist. Our approach had inevitable limitations. We were only able to search peer-reviewed studies and grey literature in English, and documents that were not widely available on the Internet, such as NGO reports, were not included. The specific needs of audience groups such as industry, the private sector, the media and the general public (who other than when involved in advocacy, have no defined role) were beyond the scope of this study. Nevertheless, this study addresses the needs of a wide range of users. An assessment of the in-depth knowledge needs of other audiences may require some adaptation of the framework.

Furthermore, while it was beyond the scope of this study, the use of the framework in conjunction with the process for identifying knowledge users’ information needs with an evidence synthesis output, would benefit from being pre-tested and pilot tested with different groups of knowledge users. Although the process currently suggests equal weighting is given to considerations of rigour, relevance, readability and resources, we would expect that different groups of policy and decision makers might emphasize different components in different contexts. For example, the primary concern for academic stakeholders might be rigour, while policy makers might consider readability and relevance to be of primary importance, and practitioners might prioritize relevance. The emphasis given to each component might lead to the adaptation and development of the framework, in order to increase its utility to different user groups.

## Conclusion

Users’ knowledge needs vary and meeting them successfully requires collaborative planning. The Framework describes the various evidence synthesis outputs identified and the process for matching users’ information needs with an appropriate output. It is intended to offer a more systematic way for users, commissioners and producers to establish a common understanding of users’ knowledge needs, and the essential characteristics to be considered when matching those needs with the most suitable output, given the resources available.

Further work would help to address the limitations of this study, such as taking the knowledge needs of other audiences into account.

## Supplementary Material

Supplementary Data
