# Supplementary material for: Taking knowledge users’ knowledge needs into account in health: an evidence synthesis framework
Source: Health Policy Plan. 2015 Aug 31;31(4):527–37. doi: 10.1093/heapol/czv079 (PMC4986240; doi:10.1093/heapol/czv079)
Supplement: Supplementary Data [file supp_31_4_527__index.html]

Taking knowledge users’ knowledge needs into account in health: an evidence synthesis framework — Taking knowledge users’ knowledge needs into account in health: an evidence synthesis framework — Supplementary Data 

# Taking knowledge users’ knowledge needs into account in health: an evidence synthesis framework

## Supplementary Data

files

- Supplementary Data - zip file
