# Supplementary material for: Taking knowledge users’ knowledge needs into account in health: an evidence synthesis framework
Source: Health Policy Plan. 2015 Aug 31;31(4):527–37. doi: 10.1093/heapol/czv079 (PMC4986240; doi:10.1093/heapol/czv079)
Supplement: Supplementary Data [file supp_czv079_suppl_data.zip › AdditionalFile1_DataAbstractionTable.docx]

**Additional File 1: Data abstraction from the documents included in the study, based on the study objectives**

| **Articles (author, year)** | **Type of evidence synthesis identified** | **Issues for consideration when planning an evidence synthesis** |
| --- | --- | --- |
| DFID Evidence Brokers (2013) | 6 evidence syntheses products | Timeframe, evidence inclusivity, format requirements |
| Saul et al. (2013) | Rapid realist reviews | 10 step methodology |
| Thomson (2013) | N/A | Evidence synthesis planning – 4 Rs for consideration |
| Chambers and Wilson (2012) | Evidence briefing | Evidence briefing checklist |
| Kastner et al. (2012) | N/A | Study protocol – to compare and map the steps for conducting different knowledge synthesis methods |
| Khangura et al. (2012) | Rapid review | 8 steps production process |
| Alliance for Health Policy and Systems Research [2011] | 3 types of short syntheses, and systematic review | N/A |
| Tricco et al. (2011) | 8 types of systematic reviews | Examples of research questions. List of steps involved in conducting a systematic review |
| Abrami, et al. (2010) | Brief review | Timeframe and scope |
| Ganann et al. (2010) | Rapid review | Methodological approaches and limitations |
| UK Civil Service (2010) | 6 evidence synthesis products | Timeframe, limitations, when useful |
| Grant and Booth (2009) | 14 review methodologies | SALSA analytical framework used to identify perceived strengths and weaknesses of each |
| Hansen and Rieper (2009) | Systematic reviews | Different methodologies used across fields |
| Lavis et al. (2009) | Policy briefs | SUPPORT tools |
| Ogilvie et al. (2009) | N/A | The role of evidence synthesis in public health research. Considers evidence synthesis to be broader than systematic reviews |
| Watt et al. (2008) | Rapid reviews and systematic reviews | Suggests transparency of methods rather than formalised methodology |
| Davies (2006) | N/A | Identifies different types of research questions and puts forward the case for different types of evidence synthesis to answer these. Also, the need for them to be written in non-technical language. |
| Arksey and O’Malley (2005) | Scoping studies | 5 stage framework for conducting a scoping study |
| Mays et al. (2005) | N/A | 4 methods for synthesising and/or analysing qualitative and quantitative data |
| Sheldon (2005) | N/A | Considers factors to be considered when producing an evidence synthesis including relevance, timing and presentation; addressing policy makers needs for different types of evidence and data |
| Petticrew et al. (2004) | N/A | Types of evidence needed by policy makers |
| Petticrew and Roberts (2003) | N/A | Typology of evidence matching different research questions to types of research that may be most useful in answering them |
| Gough and Elbourne (2002) | N/A | Systematic research syntheses need to be explicit and transparent about the method used, although methodologies may vary depending on the research question |
| Largay 2001 | N/A | Readability, rigour and relevance |
