# Supplementary figures and images for: Taking knowledge users’ knowledge needs into account in health: an evidence synthesis framework
Source: Health Policy Plan. 2015 Aug 31;31(4):527–37. doi: 10.1093/heapol/czv079 (PMC4986240; doi:10.1093/heapol/czv079)

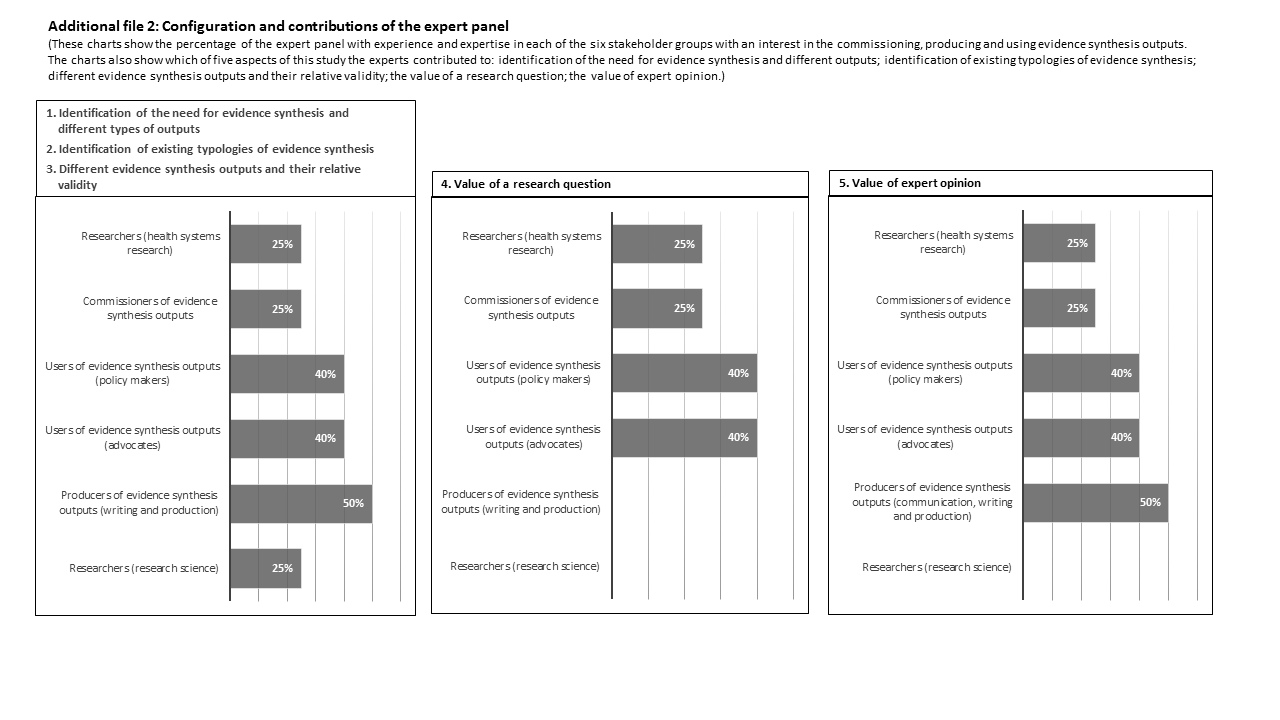

Supplement: Supplementary Data [file supp_czv079_suppl_data.zip › AdditionalFile2_ConfigurationAndContributionsOfExpertPanel.tif]
